# Alexithymia Components Are Differentially Related to Explicit Negative Affect But Not Associated with Explicit Positive Affect or Implicit Affectivity

**DOI:** 10.3389/fpsyg.2017.01758

**Published:** 2017-10-09

**Authors:** Thomas Suslow, Uta-Susan Donges

**Affiliations:** ^1^Department of Psychosomatic Medicine and Psychotherapy, University of Leipzig, Leipzig, Germany; ^2^Department of Psychiatry, Psychotherapy and Psychosomatics, Martin Gropius Krankenhaus, Eberswalde, Germany; ^3^Department of Psychiatry and Psychotherapy, Campus Charité Mitte – Universitätsmedizin, Berlin, Germany

**Keywords:** alexithymia, 20-Item Toronto-Alexithymia Scale, explicit affect, implicit affect, Implicit Positive and Negative Affect Test

## Abstract

Alexithymia represents a multifaceted personality construct defined by difficulties in recognizing and verbalizing emotions and externally oriented thinking. According to clinical observations, experience of negative affects is exacerbated and experience of positive affects is decreased in alexithymia. Findings from research based on self-report indicate that all alexithymia facets are negatively associated with the experience of positive affects, whereas difficulties identifying and describing feelings are related to heightened negative affect. Implicit affectivity, which can be measured using indirect assessment methods, relates to processes of the impulsive system. The aim of the present study was to examine, for the first time, the relations between alexithymia components and *implicit* and *explicit* positive and negative affectivity in healthy adults. The 20-item Toronto Alexithymia Scale, the Implicit Positive and Negative Affect Test and the Positive and Negative Affect Schedule (PANAS) were administered to two hundred and forty-one healthy individuals along with measures of depression and trait anxiety. Difficulties identifying feelings were correlated with explicit negative trait affect, depressive mood and trait anxiety. Difficulties describing feelings showed smaller but also significant correlations with depressive mood and trait anxiety but were not correlated with explicit state or trait affect as assessed by the PANAS. Externally oriented thinking was not significantly correlated with any of the implicit and explicit affect measures. According to our findings, an externally oriented, concrete way of thinking appears to be generally unrelated to dispositions to develop positive or negative affects. Difficulties identifying feelings seem to be associated with increased conscious negative affects but not with a heightened disposition to develop negative affects at an automatic response level.

## Introduction

Alexithymia is a cognitive-affective disturbance that is characterized by impairments in the experience, regulation and communication of emotions (Nemiah and Sifneos, 1970; Taylor and Bagby, 2000). The alexithymia construct emerged following attempts of clinicians to specify the emotional deficits of psychosomatic patients (Ruesch, 1948; Sifneos, 1973). These patients showed an unawareness of feelings or an incapacity to put into words what they were experiencing. Their associations were characterized by an absence of fantasy and a detailed recounting of circumstances and events in their environment (Nemiah and Sifneos, 1970). Alexithymia is best conceptualized as a dimensional personality trait and not as a categorical all-or-none phenomenon (Parker et al., 2008). Currently, the predominant measure of alexithymia is a self-report instrument, the 20-Item Toronto Alexithymia Scale (TAS-20, Bagby et al., 1994a), a questionnaire with satisfactory psychometric properties (Parker et al., 2003). The TAS-20 does not assess all facets of alexithymia but is focused on three main features of alexithymia: difficulties in recognizing and verbalizing emotions and an externally oriented thinking style (Taylor and Bagby, 2004). Patients with mental disorders such as panic disorder (De Berardis et al., 2007), somatoform disorders (Waller and Scheidt, 2004) or autistic disorders (Berthoz and Hill, 2005) show frequently high degrees of alexithymia.

It is an important question whether individuals who have problems in identifying and describing emotions experience less or more emotions in their everyday lives or show no apparent deviations in the frequency of experiencing emotions compared to individuals without these difficulties. It is possible, for example, that individuals who develop less frequently and weaker reactions of happiness in everyday life may tend to talk less about their positive feelings and describe more situational details or their concrete actions which are more salient to them compared to individuals with frequent and strong positive emotions. Early in alexithymia research, based on clinical observations it was hypothesized that alexithymic individuals manifest tendencies to experience more negative affects and less positive affects so that they were also described as anhedonic (Sifneos, 1987; Bagby and Taylor, 1997).

Typically, state (actual) and trait (habitual) affects are measured by means of self-report questionnaires. The Positive and Negative Affect Schedule (PANAS; Watson et al., 1988) assessing the intensity or frequency of experience of positively and negatively valenced affects has been repeatedly administered to assess state and trait affectivity as a function of alexithymia. As expected, in a sample of university students alexithymic individuals were found to endorse more negative trait affects and less positive trait affects than non-alexithymic individuals (Parker and Taylor, 1997). In a correlational study based on a community sample (*n* = 137), the total score of the TAS-20 showed positive correlations of small to medium sizes with trait measures of negative affect and negative correlations of medium to large effect sizes with trait measures of positive affect (Lundh and Simonsson-Sarnecki, 2001). In a large sample (*n* = 377) of patients presenting to primary care physicians with physical symptoms (de Gucht et al., 2004), all TAS-20 scales showed negative correlations of (small to) medium size with positive state affect. Moreover, difficulties identifying (*r* = 0.51) and describing feelings (*r* = 0.20) (but not externally oriented thinking) were associated with negative state affect. In a sample of university students (*n* = 175), difficulties identifying and describing feelings, and externally oriented thinking showed negative correlations of medium size with positive state affect and only difficulties identifying feelings (*r* = 0.21) and the TAS total score (*r* = 0.18) correlated significantly (positively) with negative state affect (Swinkels and Giuliano, 1995, study 3). However, Palmer et al. (2002) found in a general community sample (*n* = 107) only small and non-significant negative correlations between TAS-20 scales and positive affect and only a significant correlation of difficulties identifying feelings with negative affect (*r* = 0.46) (PANAS: reference period of the affect rating was the last month). In a rather large mixed sample of older adults from the general population and university students (*n* = 248), Henry et al. (2006) observed a negative correlation with positive affect only for difficulties identifying feelings (*r* = -0.21) whereas difficulties identifying feelings and difficulties describing feelings were positively correlated with negative affect (*r* = 0.25 and 0.37). In the latter study, externally oriented thinking showed a small but significant negative correlation with negative affect (*r* = -0.15) (PANAS: reference period of the affect rating here was the last week). Overall, there is evidence that the alexithymia components difficulties identifying and describing feelings and externally oriented thinking are negatively related to experiences of positive affect and that primarily difficulties identifying feelings and secondarily difficulties describing feelings are associated with experiences of negative affect. Considering that in healthy subjects positive affects are experienced far more frequently and intensely than negative affects (Zelenski and Larsen, 2000; Myrtek et al., 2005) reduced feeling of positive affects should be given more weight when examining the affective deviations in alexithymia.

Previous research on emotions in alexithymia has focused primarily on negative emotional conditions. There is ample evidence across patient and general population samples for medium relationships between difficulties identifying and describing feelings with depressive mood. In contrast, the alexithymia component externally oriented thinking shows no or only small correlations with depressive symptoms (see Li et al., 2015, for a meta-analysis). Moreover, there are findings indicating large correlations between difficulties identifying and difficulties communicating feelings and trait anxiety but no correlations between externally oriented thinking and trait anxiety (Hendryx et al., 1991; Berthoz et al., 1999). The above-mentioned results based mainly on self-report or direct measures of emotions suggest a differential association between alexithymia components and negative affect.

When direct assessment procedures such as the PANAS are administered conscious affective experiences are reported which has been termed *explicit affect*. Explicit affect is assumed to build on propositionally organized memory that is subject to conscious reflections (Quirin et al., 2009a). Instead, *implicit affect* relates to processes of the impulsive system and represents automatic activations of cognitive representations of affective experiences. The impulsive system is thought to elicit behavior through associative links and motivational orientations (Strack and Deutsch, 2004). The Implicit Positive and Negative Affect Test (IPANAT) has been constructed to assess implicit affectivity (Quirin et al., 2009a). In this indirect test, the extent of fit between mood adjectives and nonsense words has to be rated. The IPANAT is a reliable instrument for the measurement of implicit affect and captures much variance from a stable psychological disposition (Quirin and Bode, 2014). Differential patterns of correlations with concurrently administered explicit and implicit tests suggest strong convergent and discriminant validity of the IPANAT (Quirin et al., 2009a; study 3). The IPANAT has been translated now into more than 10 languages and has found wide distribution (Quirin et al., 2016).

The IPANAT predicts spontaneous psycho-physiological reactions, above and beyond self-reported affectivity. Implicit positive affect predicted low cortisol levels in everyday life (Mossink et al., 2015). Furthermore, low implicit positive affect was found to be associated with circadian cortisol release and high implicit negative affectivity was found to be related to cortisol response to acute stressors (Quirin et al., 2009b). Recently, implicit affectivity measured by the IPANAT was associated with cardiovascular activity during and after stressful tasks (van der Ploeg et al., 2016). Finally, implicit negative affect predicted neural activation in response to threat, in brain areas responsible for fear and flight behavior (Suslow et al., 2015). Thus, it seems that the IPANAT is useful in gathering information on dispositions to affective reactions in the absence of conscious self-reflection. Against this background, it is quite possible that explicit affect scores are less informative about spontaneous affective responsivity and experiences than implicit affect scores.

The aim of our study was to examine, for the first time, implicit and explicit positive and negative affectivity as a function of alexithymia in healthy adults. For this purpose, we administered the IPANAT and state and trait versions of the PANAS as well as measures of depressed mood and trait anxiety. Based on previous research with the PANAS, it was hypothesized that all alexithymia components would be negatively correlated with measures of positive affect. Moreover, it was expected that the alexithymia features difficulties identifying feelings and difficulties describing feelings would be positively correlated with measures of negative affect. In the light of the foregoing considerations, it could be derived that the alexithymia facet externally oriented thinking is not related to negative affect but negatively associated to positive affect. That is, individuals high on externally oriented thinking might be characterized by a decreased disposition to develop positive emotions. In contrast, especially the alexithymia component difficulties identifying feelings appears to come along with an increased disposition to develop negative emotions. We expected that the healthy individuals in our study would report more positive state and trait affect than negative state and trait affect. Moreover, it was hypothesized that our subjects would manifest more implicit positive affect than implicit negative affect. We controlled verbal intelligence of participants because it has been found to be associated with alexithymia (e.g., Lamberty and Holt, 1995; Montebarocci et al., 2011).

## Materials and Methods

### Participants

Two hundred and forty-one volunteers (192 women) with a mean age of 24.76 years (*SD*: 3.85) and a mean school education of 12.37 years (*SD*: 0.75) participated in the present study. All participants were native German speakers. They were free of any lifetime history of psychiatric or neurological disorders. According to self-report all participants did not use psychotropic medication. Participants were recruited via public notices. Notices with a short description of the study, the exclusion criteria and a contact telephone number were posted in several locations on the campus of the University (e.g., libraries and canteens). More than 92% of participants were university students from various disciplines.

Men did not differ from women on any of the variables examined in our study (i.e., alexithymia components, affect scores (IPANAT, PANAS, BDI-II and STAI), verbal intelligence, and education), except age. Men (mean age: 26.53; *SD*: 3.70) were about 2 years older than women (mean age: 24.31; *SD*: 3.77) [*t*(239) = 3.70, *p* < 0.001].

The present study was carried out according to the Declaration of Helsinki (World Medical Association, 2013). Written informed consent was obtained from all study participants prior to data collection. The study was approved by the competent ethics committee of the Medical Faculty at the University of Leipzig.

### Psychometric Instruments

Alexithymia was measured by the 20-Item Toronto-Alexithymia Scale (TAS-20; German version: Bach et al., 1996). The TAS-20 consists of three subscales: Difficulties identifying feelings (DIF; consisting of 7 items), Difficulties describing feelings (DDF; consisting of 5 items), and Externally oriented thinking (EOT; consisting of 8 items). Items are rated on a 5-point Likert scale (from 1 = “strongly disagree” to 5 = “strongly agree”). Validation studies have revealed adequate convergent and discriminant validity, internal consistency, and reliability for the TAS-20 (Bagby et al., 1994a,b; Parker et al., 2003). In the present sample, mean scores for Difficulties identifying feelings, Difficulties describing feelings, Externally oriented thinking, and the total scale were 14.32 (*SD* = 4.67), 11.48 (*SD* = 3.84), 15.62 (*SD* = 4.00), and 41.42 (*SD* = 9.03), whereas Cronbach‘s alphas for the scales were 0.79, 0.75, 0.62, and 0.79 (which were very similar to the coefficients reported by Bagby et al. (1994a) for a sample of university students: 0.79, 0.75, 0.66, and 0.80).

The *Implicit Positive and Negative Affect Test* (IPANAT, Quirin et al., 2009a) was applied to assess implicit positive and negative affectivity. The IPANAT measures affect indirectly by asking to evaluate to what extent artificial words express certain moods. Six artificial words (e.g., TALEP and VIKES) are presented along with three positive (happy, cheerful, and energetic) and three negative mood words (inhibited, helpless, and tense). Assessments are made on a 4-point scale [from 1 (*doesn’t fit at all*) to 4 (*fits very well*)]. Factor analysis has yielded two orthogonal factors that can be interpreted as positive affect and negative affect (Quirin et al., 2009a, 2016). For each scale, Cronbach’s alphas were found to be above.80, whereas 1-year test-retest reliability was about 0.60. The latter finding suggests that the IPANAT captures much variance from a stable affective disposition. In our study, the mean item score for the IPANAT-PA was 2.33 (*SD*: 0.33) and that for the IPANAT-NA was 1.88 (*SD*: 0.46). These IPANAT scores were very similar to those reported previously for German university students (Quirin et al., 2009a). In the present study, Cronbach’s alpha was 0.79 for implicit PA and 0.76 for implicit NA.

The Positive and Negative Affect Schedule (PANAS; German version: Krohne et al., 1996) was administered to measure state and trait positive affect (PA) and negative affect (NA). The PANAS consists of 10 negative and 10 positive mood questions, rated on a five-point scale (1 = not at all, 5 = extremely). The mean PANAS-PA trait score was 36.67 (*SD*: 5.35) and the mean PANAS-NA trait was 16.29 (*SD*: 5.01) in our sample. Moreover, the mean PANAS-PA state was 33.84 (*SD*: 5.91) and the mean PANAS-NA state was 13.21 (*SD*: 3.96). In our study, Cronbach‘s alpha was 0.74 for the PANAS-PA S (state) and 0.79 for the PANAS-NA S. Moreover, Cronbach‘s alpha was 0.81 for the PANAS-P T (trait) and 0.85 for the PANAS-NA T.

Depressed mood and trait anxiety were measured by administering the Beck-Depression Inventory (BDI-II; German version: Hautzinger et al., 2009) and the State-Trait-Anxiety Inventory trait version (STAI; German version: Laux et al., 1981). In our sample, the mean BDI-II score was 7.08 (*SD* = 4.99) and the mean STAI-Trait score was 38.06 (*SD* = 9.00). Cronbach‘s alpha was 0.79 for the BDI-II and 0.88 for the STAI in our sample.

Verbal intelligence was assessed by means of the Mehrfachwahl-Wortschatz-Intelligenztest (MWT-B), a multiple-choice test using artificial and existent vocabulary of the German language (Lehrl, 2005). The MWT-B consists of 37 items and has no time restrictions. In our sample, the mean MWT-B IQ-score was 113.49 (*SD* = 11.31).

### Statistical Analyses

If not otherwise stated, statistical data analyses were conducted by means of the Statistical Package for the Social Sciences (SPSS), version 24.0. Paired samples *t*-tests were conducted to determine differences between positive and negative affect as assessed by implicit (IPANAT) and explicit affectivity measures (PANAS state and PANAS trait). In these *t*-tests a Bonferroni-corrected statistical significance threshold of *p* < .017 was applied (α = 0.05/3). Product-moment correlation analyses were performed to investigate the relationships between implicit and explicit affectivity and the associations of alexithymia with implicit and explicit affectivity, verbal intelligence, education, and age. Since the correlations of alexithymia scores (Difficulties identifying feelings, Difficulties describing feelings, Externally oriented thinking and TAS-20 total score) with implicit and explicit (positive and negative) affectivity (IPANAT-PA, IPANAT-NA, PANAS-S PA, PANAS-S NA, PANAS-T PA and PANAS-T NA), depression (BDI-II), trait anxiety (STAI), verbal intelligence (MWT-B), education and age were central in the present paper and included in total 44 calculations we administered a Bonferroni-corrected statistical significance threshold of *p* < 0.001 in the correlational analyses [α = 0.05/(4 × 11) = 0.0011]. Steiger’s Z-test was used to compare (significant) correlation coefficients (applying formulas as implemented in Lee and Preacher (2013)). Partial correlation analysis was conducted to illustrate the correlation between an alexithymia component and an affect score when other relevant alexithymia components were controlled.

### General Procedure

At the beginning of the study, demographic data were registered and participants were given the TAS-20, MWT-B, IPANAT, PANAS-S, BDI-II, STAI, and PANAS-T in a fixed order. Participants were tested individually and received a financial compensation for taking part in the study.

## Results

### Relationships between Implicit and Explicit Affectivity

In our sample, implicit negative affect did not correlate with explicit positive and negative affectivity (trait or state) as assessed by the PANAS, depressive mood (BDI-II), or trait anxiety (STAI-T). Implicit positive affect was only found to be correlated with explicit positive trait affect (*r* = 0.22; *p* ≤ 0.001).

### Comparison between Positive and Negative Affect As Assessed by IPANAT and PANAS State and Trait

According to the results of dependent *t*-tests, study participants had higher implicit positive than implicit negative affect scores [*t*(240) = 12.97, *p* < 0.001]. Moreover, participants reported more explicit positive state affect than explicit negative state affect [*t*(240) = 44.94, *p* < 0.001] and more explicit positive trait affect than explicit negative trait affect [*t*(240) = 41.85, *p* < 0.001].

### Relationships between Alexithymia Scores

As could be expected, the total score of the TAS-20 showed significant correlations with all TAS-20 subscales (Difficulties identifying feelings: *r* = 0.74, *p* < 0.001; Difficulties describing feelings: *r* = 0.82, *p* < 0.001; Externally oriented thinking: *r* = 0.61; *p* < 0.001). The alexithymia component Difficulties identifying feelings was significantly correlated with Difficulties describing feelings (*r* = 0.47; *p* < 0.05) but not with Externally oriented thinking: *r* = 0.05; *p* = 0.46). Finally, Difficulties describing feelings was significantly correlated with Externally oriented thinking: *r* = 0.33; *p* < 0.001).

### Relationships of Alexithymia Scores with Implicit and Explicit Affectivity

Product-moment correlation analysis showed significant positive correlations of Difficulties identifying feelings with explicit negative trait affect (PANAS), depressive mood (BDI-II) and trait anxiety (STAI) (see **Table 1**). The alexithymia component Difficulties describing feelings was significantly positively correlated with depressive mood (BDI-II) and trait anxiety (STAI).

**Table 1:** Product moment correlations between alexithymia components (20-Item Toronto Alexithymia Scale) and implicit and explicit affectivity measures, intelligence, education and age (N = 241).

Variable	DIF	DDF	EOT	TS
IPANAT-PA	-0.02	-0.03	-0.02	-0.03
IPANAT-NA	0.03	0.00	-0.02	0.01
PANAS-S PA	0.00	-0.03	-0.12	-0.07
PANAS-S NA	0.19	0.07	-0.08	0.09
PANAS-T PA	-0.08	-0.06	-0.06	-0.10
PANAS-T NA	0.38^∗∗∗^	0.11	-0.03	0.23^∗^
Depression (BDI-II)	0.41^∗∗∗^	0.22^∗^	-0.10	0.26^∗∗^
Trait anxiety (STAI trait)	0.48^∗∗∗^	0.23^∗^	-0.09	0.29^∗∗∗^
Intelligence (MWT-B IQ)	-0.18	-0.14	-0.07	-0.18
Education	-0.03	0.00	-0.04	-0.03
Age	-0.19	-0.13	0.00	-0.15

*^∗^*p* ≤ 0.001 (two-tailed), ^∗∗^*p* ≤ 0.0001 (two-tailed), ^∗∗∗^*p* ≤ .00001 (two-tailed). DIF, Difficulties Identifying Feelings scale; DDF, Difficulties Describing Feelings scale; EOT, Externally Oriented Thinking scale; TS, Total score of the 20-Item Toronto-Alexithymia Scale; IPANAT-PA, Positive affect scale – Implicit Positive and Negative Affect Test; IPANAT-NA, Negative affect scale – Implicit Positive and Negative Affect Test; PANAS-S PA, Positive affect scale of the Positive and Negative Affect Schedule state version; PANAS-S NA, Negative affect scale of the Positive and Negative Affect Schedule state version; PANAS-T PA, Positive affect scale of the Positive and Negative Affect Schedule trait version; PANAS-T NA, Negative affect scale of the Positive and Negative Affect Schedule trait version; BDI-II, Beck-Depression Inventory; STAI-trait, State-Trait Anxiety Inventory, trait version; MWT-B IQ, intelligence quotient as assessed by the Mehrfach-Wahl-Intelligenztest version B.*

According to Steiger’s *Z-*tests the correlation coefficient of Difficulties identifying feelings and depression was significantly higher than that of Difficulties describing feelings and depression (*Z* = 3.08, *p* < 0.01). Moreover, the correlation between Difficulties identifying feelings and trait anxiety was significantly higher than that between Difficulties describing feelings and trait anxiety (*Z* = 4.44, *p* < 0.001). The correlations of Difficulties identifying feelings with depression and trait anxiety remained significant when adjusting for Difficulties describing feelings (*r_p_* = 0.36 and *r_p_* = 0.44; *p*s < 0.001). However, the correlations of Difficulties describing feelings with depression and trait anxiety became non-significant when controlling for Difficulties identifying feelings (*r_p_* = 0.03 and *r_p_* = -0.02).

Externally oriented thinking was not significantly correlated with any of the implicit and explicit affect measures. All correlations coefficients of Externally oriented thinking with implicit and explicit affect were negative (but non-significant) (see **Table 1** for details). The total score of the TAS-20 was found to be significantly positively correlated with explicit negative trait affect, depressive mood and trait anxiety.

### Relationships of Alexithymia Scores with Intelligence, Age and Education

Product-moment correlation analysis showed no significant correlations between Difficulties identifying feelings, Difficulties describing feelings, Externally oriented thinking and the total TAS-20 score on the one hand and verbal intelligence on the other hand. There were also no significant correlations of alexithymia scores with age and years of school education (see **Table 1** for details).

## Discussion

The primary aim of the present study was to investigate the relationship of the personality trait alexithymia with implicit and explicit positive and negative affectivity in healthy adults. We applied an indirect (IPANAT) and direct tests (PANAS, BDI-II and STAI) to assess implicit and explicit affectivity in a sample of young adults and conducted correlation analyses for all alexithymia components of the TAS-20. As hypothesized, the participants of our study reported much more positive state and trait affect than negative state and trait affect. This is not surprising but confirms the observation that there is a clear preponderance of positive affect in healthy people (Diener and Diener, 1996; Zelenski and Larsen, 2000). Importantly, a prevalence of positive affect was also observed at an implicit affect level: our study participants were characterized by higher implicit positive than implicit negative affect scores. As could be expected, implicit positive affect showed a small to medium correlation with explicit positive trait affect in our sample.

Contrary to our hypothesis, none of the alexithymia components was negatively correlated with positive affect. We found no correlations between the different facets of alexithymia and implicit or explicit (state and trait) positive affect. Thus, our findings contrast with those of previous research based on a student sample (Swinkels and Giuliano, 1995, study 3) or an outpatient sample (de Gucht et al., 2004) but are similar to those of other studies based primarily on samples of the general population suggesting no (Palmer et al., 2002) or only a negative correlation of difficulties identifying feelings and positive affect (Henry et al., 2006). The small correlation between externally oriented thinking and explicit positive state affect (*r* = -0.12) was the highest observed in the present sample between alexithymia components and positive affect scales. According to our findings, there is no substantial relationship between the facets of alexithymia as assessed by the TAS-20 and the actual or habitual experience of positive affect. It is possible that individuals with clinically relevant degrees of alexithymia might manifest tendencies to experience less positive affects and might thus appear anhedonic. However, our null findings challenge the idea that alexithymic features go along with a reduced experience of positive affect, at least in healthy and educated young adults.

The present results confirm our hypothesis that the alexithymia components difficulties identifying feelings and difficulties describing feelings are positively associated with negative affect. We observed significant positive correlations of difficulties identifying feelings with explicit negative trait affect (PANAS), depressive mood and trait anxiety. The alexithymia component difficulties describing feelings was not found to be significantly correlated with explicit negative state and trait affect as assessed by the PANAS but correlated positively with depressive mood and trait anxiety. However, when controlling for difficulties identifying feelings difficulties describing feelings was not any more significantly related to depressive mood and trait anxiety. According to our findings, the alexithymia component difficulties identifying feelings is more strongly associated with negative affect than difficulties describing feelings which is consistent with results from other studies administering the PANAS (Swinkels and Giuliano, 1995; Palmer et al., 2002; de Gucht et al., 2004). Our data are also in line with previous findings (Berthoz et al., 1999) indicating stronger relations of difficulties identifying feelings with depression and trait anxiety compared to those of difficulties describing feelings. In clear contrast to difficulties identifying feelings, externally oriented thinking was not related to any of the negative affectivity measures administered in our study which is consistent with the results of previous research (Hendryx et al., 1991; Swinkels and Giuliano, 1995; Berthoz et al., 1999; Palmer et al., 2002; de Gucht et al., 2004).

An important focus of the present investigation was to examine for the first time implicit affectivity as a function of alexithymia. According to our data, none of the alexithymia components of the TAS-20 was related to implicit positive or implicit negative affect. Thus, administering an indirect measure of affectivity we found no evidence for correlations between alexithymia facets and implicit affect. Implicit affect is thought to be related to processes of the impulsive system (Quirin et al., 2009a). It has been found to predict endocrine stress reactions in everyday life as well as cardiovascular and endocrine responses to acute stressors (Quirin et al., 2009b; Mossink et al., 2015; van der Ploeg et al., 2016) as well as brain activation to subtle threat stimuli (Suslow et al., 2015). It should be noted that for externally oriented thinking and difficulties describing feelings non-correlations with positive and negative affectivity could be observed for the indirect (IPANAT) as well as the direct measure (PANAS). Based on our data it can be concluded that in healthy and educated adults the alexithymia facet externally oriented thinking is independent from dispositions to develop positive or negative affects.

Difficulties identifying feelings was also not correlated with implicit or explicit positive affect in our sample but it showed substantial correlations with explicit negative trait affect (and depression and trait anxiety) but not with implicit negative affect. Thus, it can be concluded that difficulties identifying feelings seem to be associated with an increased conscious experience of negative affects but not with a heightened disposition to develop negative affects at an automatic response level. Lumley (2000) proposed an explanation for the overlap of difficulties identifying feelings and depression suggesting that negative affect could promote critical perceptions of the self thereby increasing the probability that individuals report that they are poor at detecting their emotions. As has been pointed out above, implicit affectivity has been found to predict psychophysiological response to stressors and threat (Quirin et al., 2009b; Mossink et al., 2015; Suslow et al., 2015). It is interesting to note in this context that research on the psychophysiological responsivity to stressors or aversive stimuli in alexithymia has not produced clear results. Previous research has found hyper-responsivity (e.g., Martin and Pihl, 1986; Infrasca, 1997), hyporesponsivity (e.g., Linden et al., 1996; Pollatos et al., 2008) or non-significant changes (e.g., Friedlander et al., 1997) in physiological parameters in response to stressors in alexithymic compared to non-alexithymic individuals. Thus, all in all there appears not to exist a robust (positive) relationship between alexithymia and spontaneous physiological reactivity to stressors or aversive stimuli.

The constellation of high explicit negative affect and normal implicit negative affect in individuals with difficulties identifying feelings could alternately be explained by deficits in down-regulating negative affective experiences. This means, even though these individuals are characterized by a normal (not heightened) disposition to develop negative affects at an automatic response level they could have problems in controlling negative affects and therefore consciously experience and report these affects for a longer time. According to Lumley (2000), in persons with difficulties in identifying feelings because of their limited ability to regulate and resolve negative affects negative affectivity remains unmodulated yielding a chronic, undifferentiated dysphoria (see also Taylor et al., 2016).

The exclusive use of a self-report measure to assess alexithymia could be criticized as a limitation of our study. Concerns have been raised that individuals with impaired affect awareness can accurately rate themselves on this lack of awareness on a self-report scale (e.g., Lane et al., 2015). However, it is conceivable that high alexithymic individuals get negative feedback from the social environment about their deficits in perceiving, feeling and communicating emotions, and their superficial ways of thinking and talking. For example, the TAS-20 item “*People tell me to describe my feelings more*” which assesses difficulties describing feelings refers to a critical comment or request of others (Bagby et al., 1994a). Against this background, alexithymic individuals might become aware of and be able to report on their own inabilities due to the integration of interpersonal feedback into their self-concept (Günther et al., 2016). Interestingly, findings from recent studies on deficits in affect perception in alexithymia in which self-report and objective measures were administered indicate that self-reported alexithymia (i.e., TAS-20) could be a better predictor of affect recognition performance than observer-rated alexithymia (Ihme et al., 2014; Brandt et al., 2015).

To sum up, the data of the present investigation suggest that when examining the relationship between alexithymia and affectivity one should not focus on total or summary scores but should take into consideration the different facets of alexithymia and administer direct as well as indirect measures of affectivity. Alexithymic characteristics and experience of affects appear to be largely independent of each other. In our study, alexithymia components were found to be differentially related to explicit negative affect but not associated with explicit positive affect or implicit affectivity. Based on our findings, it can be concluded that in healthy young adults an externally oriented, concrete way of thinking is unrelated to dispositions for positive or negative affects. Instead, difficulties identifying feelings appear to be associated with an increased conscious experience of negative affects but not with a heightened disposition for negative affects at an automatic response level. Clearly, the generalizability of our findings is limited, as we recruited primarily female university students as participants. Therefore, it is necessary that future research on this topic includes also samples of men, elderly people and individuals with less education. It appears promising to administer the IPANAT in psycho-physiological research on affective responsivity in alexithymia along with direct measures of affectivity. Longitudinal experiments examining the recovery from (implicit and explicit) negative affects over time as a function of alexithymia components could give more detailed insights into the emotion regulation deficits associated with difficulties identifying feelings.

## Author Contributions

TS and U-SD contributed equally to the conception and design of the study as well as to the analysis and interpretation of data. TS organized collection of data. TS wrote the first draft of the manuscript and U-SD made critical revisions. Both authors approved the final version of the manuscript for publication.

## Conflict of Interest Statement

The authors declare that the research was conducted in the absence of any commercial or financial relationships that could be construed as a potential conflict of interest.
